# Identifying the best reference gene for RT-qPCR analyses of the three-dimensional osteogenic differentiation of human induced pluripotent stem cells

**DOI:** 10.1016/j.bonr.2024.101816

**Published:** 2024-11-17

**Authors:** Masakazu Okamoto, Yusuke Inagaki, Kensuke Okamura, Yoshinobu Uchihara, Kenichiro Saito, Akihito Kawai, Munehiro Ogawa, Akira Kido, Eiichiro Mori, Yasuhito Tanaka

**Affiliations:** a Department of Rehabilitation Medicine, Nara Medical University, Kashihara, Nara, Japan; b Department of Orthopaedic Surgery, Nara Medical University, Kashihara, Nara, Japan; c Department of Orthopaedic Surgery, Higashiosaka City Medical Center, Higashiosaka, Osaka, Japan; d Department of Future Basic Medicine, Nara Medical University, Kashihara, Nara, Japan

**Keywords:** hiPSCs, Osteogenic differentiation, Reference gene, Regenerative medicine, RT-qPCR

## Abstract

Reverse transcription quantitative real-time polymerase chain reaction (RT-qPCR) is an essential tool for gene expression analysis; choosing appropriate reference genes for normalization is crucial to ensure data reliability. However, most studies on osteogenic differentiation have had limited success in identifying optimal reference genes. To the best of our knowledge, no optimal reference genes in three-dimensional (3D) osteogenic differentiation culture experiments using human induced pluripotent stem cells (hiPSCs) have been identified. In this study, we aimed to identify stable reference genes that could be used for normalization in gene expression analyses during the 3D osteogenic differentiation of hiPSCs using an atelocollagen sponge as a scaffold. Four algorithms—ΔCt, BestKeeper, NormFinder, and geNorm—were used to evaluate the stability of 14 candidate reference genes. Genes encoding TATA box-binding protein, hypoxanthine phosphoribosyltransferase 1, and 14–3-3 protein zeta polypeptide were identified as the most stable reference genes. In comparison, conventionally used reference genes (beta-2 microglobulin and beta-actin genes) ranked among those with low stability. We also demonstrated the successful 3D osteogenic differentiation of hiPSCs on atelocollagen sponge. Our findings provide valuable insights for reference gene selection and bone tissue regeneration from hiPSCs, which could improve the treatment prospects for bone defects and other similar conditions in regenerative medicine.

## Highlights

• We aimed to identify stable reference genes for 3D osteogenic differentiation of hiPSCs that could be used for normalization
• Stability was evaluated using 4 algorithms—ΔCt, BestKeeper, NormFinder, and geNorm
• *TBP*, *HPRT1*, and *YWHAZ* were the most stable reference genes
• Conventionally used reference genes (*B2M* and *ACTB*) exhibited low stability
• Our findings can aid reference gene selection and bone tissue regeneration

## 1 Introduction

Reverse transcription quantitative real-time polymerase chain reaction (RT-qPCR) is an important and convenient technique for monitoring relative changes in gene expression. Although RT-qPCR is widely used for gene expression analysis, several factors can affect its accuracy. Variability in sample preparation, RNA quality, reverse transcription efficiency, and experimental conditions can lead to considerable fluctuations that may result in erroneous conclusions. Therefore, normalization is necessary to ensure data reliability (VanGuilder et al., 2008), and this is clearly defined in the guidelines of Minimum Information for the Publication of Quantitative Real-Time PCR Experiments (MIQE) (Bustin et al., 2009). The reference gene chosen for normalization should exhibit stable expression throughout experiments and across different groups. However, inappropriate normalization remains one of the drawbacks of qPCR. Furthermore, this issue is often underreported in gene expression studies (Hasler et al., 2020).

A common approach for normalization is to compare the expression of the target gene with that of an endogenous control gene within the same sample and experimental conditions. Housekeeping genes are essential for fundamental cellular functions and are conventionally used as reference genes (De Spiegelaere et al., 2015) because they are constitutively expressed, and their expression is expected to be stable under various physiological and experimental conditions. However, recent studies have revealed that the expression levels of housekeeping genes vary depending on the chosen gene, cell type, and experimental conditions. Therefore, to accurately analyze the expression of target genes, it is crucial to validate the stability of normalized genes in target cells (Okamura et al., 2020).

In this study, we focused on human induced pluripotent stem cells (hiPSCs), which demonstrate potential in bone tissue engineering owing to their long-term self-renewal and multilineage differentiation abilities (Takahashi and Yamanaka, 2006). In particular, their ability for indefinite self-renewal makes them a promising candidate for treating bone defects and studying the mechanisms underlying bone diseases (Kidwai et al., 2023). In experiments on osteogenic differentiation using hiPSCs, it is important to consider optimal reference genes to avoid the misinterpretation of the RT-qPCR results. In a previous study conducted in a two-dimensional (2D) setting, the gene encoding TATA box-binding protein (*TBP*) was found to be the most stable endogenous control gene (Okamura et al., 2020). Although monolayer cell culture is a convenient method for studying cell differentiation mechanisms, it lacks the three-dimensional (3D) characteristics inherent to tissues. Therefore, in tissue engineering, it is necessary to replicate the 3D microenvironment using natural or synthetic materials. Furthermore, 3D culture can influence cell morphology, proliferation, and gene expression more strongly than 2D culture, necessitating the identification of robust reference genes for quantitative gene expression analyses (Hasler et al., 2020). The objective of this study was to identify the most stably expressed reference genes for gene expression analysis during osteogenic differentiation induction experiments using hiPSCs cultured in a 3D environment.

## 2 Methods

### 2.1 Human iPS cell lines and induction of 3D osteogenic differentiation

The commercially available Cellartis Human iPS Cell Line 12 (Takara Bio Inc., Kusatsu, Japan) was used in this study. The cells were seeded in wells coated with Matrigel basement membrane matrix (Corning Inc., Corning, NY, USA), which is suitable for feeder-free culture, and maintained in Essential 8 (E8) medium (Thermo Fisher Scientific Inc., Waltham, MA, USA), an undifferentiated state maintenance medium. The medium was changed daily, and once the cells reached confluence (approximately 80 %), they were detached using Versene (Thermo Fisher Scientific) and passaged. The passaged cells were used in subsequent osteogenic differentiation experiments. A commercial atelocollagen sponge (Mighty, KKN-CSM-50; Koken Co., Tokyo, Japan) was used as the scaffold material. Prior to use, the sponge was soaked in E8 medium for 24 h. Each sponge was placed in a well of a 24-well plate, and the cell suspension (50 μL; containing 3.0 × 10^4^ cells) was carefully pipetted onto the upper surface of the sponge. After seeding, the cells were cultured in E8 medium for 3 days; the end of this time point was defined as day 0. From day 0, the medium was changed to Dulbecco's modified Eagle's medium (DMEM; Nacalai Tesque Inc., Kyoto, Japan) supplemented with 15 % fetal bovine serum (GE Healthcare Life Sciences, Logan, UT, USA), 1 % non-essential amino acids (Sigma-Aldrich Inc., St. Louis, MO, USA), 1 % antibiotic-antimycotic solution (Nacalai Tesque Inc.), and β-mercaptoethanol (100 μM; Nacalai Tesque Inc.) to induce osteogenic differentiation. The medium was changed daily. Starting from day 3, the above medium was supplemented with β-glycerophosphate (10 mM; Nacalai Tesque Inc.), l-ascorbic acid (50 μg/mL; FUJIFILM Wako Pure Chemical Corporation, Osaka, Japan), dexamethasone (10 nM; Sigma-Aldrich), and 1,25-(OH)_2_ vitamin D3 (50 nM; FUJIFILM Wako) to induce osteogenic differentiation. The culture medium was changed every 2 days for 28 days. Samples were collected on days 0, 7, 14, 21, and 28.

### 2.2 Preparation of frozen sections

To create frozen sections, the atelocollagen sponge was washed with phosphate-buffered saline (PBS) (FUJIFILM Wako) and fixed at 25 °C for 15 min in 4 % paraformaldehyde buffer (FUJIFILM Wako). After washing with PBS, the samples were dehydrated overnight at 4 °C using 30 % sucrose prepared in PBS. Subsequently, they were embedded in optimal cutting temperature compound (Sakura Finetek Japan, Tokyo, Japan) and rapidly frozen in liquid nitrogen. These samples were stored at −80 °C until further analysis, and 15 μm-thick sections were prepared using a cryostat (Leica CM1950; Leica Biosystems, Wetzlar Germany), as previously described (Kawamoto and Kawamoto, 2014).

### 2.3 Alizarin red S staining

To evaluate calcium deposition within the sponge on day 28, the sections were stained with Alizarin Red S (Nacalai Tesque Inc.). Calcium deposits were stained red.

### 2.4 Fluorescent immunostaining

Briefly, iPS cells (3.0 × 10^4^ cells) were seeded on an atelocollagen sponge and cultured. On day 28, immunolocalization of type I collagen was examined. The sections were rinsed several times with PBS and incubated for 1 h in 3 % bovine serum albumin to block nonspecific binding. These sections were incubated with a primary antibody against type I collagen alpha 1 (COL1A1; diluted 1:100; 322-COLTt, PhosphoSolutions, Denver, CO, USA) at 37 °C in a humid chamber for 1 h. Subsequently, the sections were incubated at 25 °C for 1 h with the secondary antibody Donkey Anti-Rabbit IgG H&L (Alexa Fluor® 488; diluted 1:1000; Thermo Fisher Scientific). The cell nuclei were counterstained with SlowFade Gold Antifade Mountant and DAPI (Thermo Fisher Scientific). The sections were observed under a confocal laser scanning microscope, LSM880 (Carl Zeiss, Japan).

### 2.5 SEM

Samples with and without cell seeding were collected on days 0, 7, and 28 of culture and observed using SEM. The samples were fixed in phosphate-buffered 2 % glutaraldehyde (Electron Microscopy Sciences, PA, USA) and post-fixed in 2 % osmium tetroxide (Heraeus Chemicals, South Africa) for 2 h in an ice bath. The specimens were dehydrated in a graded ethanol series (Nacalai Tesque Inc.) and freeze-dried using t-butyl alcohol (Kanto Chemical Co., Inc., Tokyo, Japan). The dried specimens were coated using an osmium plasma ion coater (Nippon Laser and Electronics Lab., Nagoya, Japan) and observed using SEM (JSM-7500F at 5 kV; JEOL Ltd., Tokyo, Japan).

### 2.6 Gene expression analysis

For total RNA extraction, 1 mL of TRIzol (Thermo Fisher Scientific) was added to a 5-mL tube along with the sponge. The mixture was then homogenized using a handheld microhomogenizer and incubated at 25 °C for 10 min. After centrifugation at 1500 ×*g* for 10 min at 4 °C, the supernatant was transferred to a QIA shredder spin column (Qiagen Inc., Valencia, CA, USA) and centrifuged again at 17,700 ×*g* for 2 min at 25 °C. The obtained filtrate was transferred to a new 1.5 mL-microcentrifuge tube and mixed thoroughly with 200 μL chloroform. The mixture was then incubated at 25 °C for 5 min. After centrifugation at 17,700 ×*g*, the upper aqueous layer was transferred to a new 1.5 mL-microcentrifuge tube, and an equal volume of 70 % ethanol was added to it. The samples were mixed well and transferred to an RNeasy Micro Kit Spin Column (Qiagen Inc.). Total RNA was recovered following the procedures outlined in the product manual and treated with DNase to prevent genomic DNA contamination. A portion of the obtained solution was diluted 20-fold with Tris-ethylenediaminetetraacetic acid buffer (pH 8.0; Nippon Gene, Tokyo, Japan) and its absorbance at 230, 260, and 280 nm was measured using an Ultrospec 3000 spectrophotometer (Biochrom Ltd., Cambridge, England) to assess RNA purity and to determine its concentration. An RNA template (1 μg) was prepared, and reverse transcription was performed using the iScript Advanced cDNA Synthesis Kit (Bio-Rad Laboratories, Hercules, CA, USA), following the manufacturer's instructions. RT-qPCR was performed using the StepOnePlus Real-Time PCR System (Applied Biosystems, Foster City, CA, USA).

### 2.7 Quantitative real-time polymerase chain reaction

The commercially available Reference Genes H96 (Bio-Rad Laboratories) was used to compare the multiple candidate reference genes. The assay IDs for the 14 human reference gene candidates evaluated in this study are listed in Table 1. The Reference Genes H96 allowed the assessment of genomic DNA contamination, cDNA quality, PCR efficiency, RNA quality, and reverse transcription efficiency. In each well, a 20 μL reaction mixture was prepared, consisting of 10 μL of 2× SsoAdvanced universal SYBR Green Supermix (Bio-Rad), 1 μL of cDNA template (25 ng), and 9 μL of nuclease-free water. The reaction steps included an initial activation at 95 °C for 2 min, followed by 40 cycles of denaturation at 95 °C for 5 s, and annealing and extension at 60 °C for 30 s. Subsequently, melting curve analysis was performed. For analyzing the expression of marker genes related to undifferentiated, osteoblast-differentiated, and osteocyte-differentiated states, internal control genes were selected based on the candidate reference genes listed in Table 1, and RT-qPCR was performed using the PrimePCR SYBR Green Assay (Bio-Rad) or TaqMan Gene Assay (Applied Biosystems). For the former, the reaction mixture included 5 μL of 2× SsoAdvanced universal SYBR Green Supermix, 0.5 μL of 20× SYBR Green Assay, 1 μL of cDNA template (25 ng), and 3.5 μL of nuclease-free water per well, with a total volume of 10 μL dispensed for each sample. The reaction conditions were the same as those used for the Reference Genes H96. For the latter, the reaction mixture included 10 μL of 2× TaqMan Fast Universal PCR Master Mix, 1 μL of 20× Gene Expression Assay, 2 μL of cDNA template (50 ng), and 7 μL of nuclease-free water per well, with a total volume of 20 μL dispensed into each sample. The reaction conditions involved an initial activation at 95 °C for 20 s, followed by 40 cycles of denaturation at 95 °C for 1 s and annealing and extension at 60 °C for 20 s.

**Table 1:** Candidate reference genes validated in the present study.

Gene symbol	Gene name	Function	Assay ID (Bio-Rad)	Amplicon length (bp)	Product efficiency
*ACTB*	Actin, beta	Cytoskeletal structural protein	qHsaCED0036269	62	2.03
*B2M*	Beta-2-microglobulin	Cytoskeletal protein involved in cell locomotion	qHsaCID0015347	123	1.98
*G6PD*	Glucose-6-phosphate dehydrogenase	Involved in glucose metabolism; promotes NADPH generation	qHsaCED0001353	137	2.05
*GAPDH*	Glyceraldehyde-3-phosphate dehydrogenase	Glycolytic enzyme	qHsaCED0038674	117	1.97
*GUSB*	Glucuronidase, beta	Hydrolyzes glucuronide bonds in lysosomes	qHsaCID0011706	79	2.03
*HMBS*	Hydroxymethylbilane synthase	Part of the heme synthesis pathway; enhances heme production	qHsaCID0038839	137	1.98
*HPRT1*	Hypoxanthine phosphoribosyltransferase 1	Involved in the metabolic salvage of purines in mammals	qHsaCID0016375	90	2.05
*PGK1*	Phosphoglycerate kinase 1	ATP-generating enzyme	qHsaCED0003721	74	1.81
*RPL13A*	Ribosomal protein L13a	Structural component of the large 60S ribosomal subunit	qHsaCED0020417	118	1.99
*RPLP0*	Ribosomal protein, large, P0	Ribosomal protein	qHsaCED0038653	63	1.95
*RPS18*	Ribosomal protein S18	Protein synthesis	qHsaCED0037454	67	1.94
*TBP*	TATA box-binding protein	General RNA polymerase II transcription factor	qHsaCID0007122	120	2.01
*TFRC*	Transferrin receptor	Takes up iron from transferrin at the cell membrane	qHsaCID0022106	102	1.96
*YWHAZ*	14–3-3 protein, zeta polypeptide	Signal transduction	qHsaCID0013897	175	1.96

### 2.8 Data analysis

To select suitable reference genes for the 3D osteogenic differentiation induction experiments involving hiPSCs, Ct values, PCR efficiency, and relative expression values based on candidate reference genes obtained using RT-qPCR were analyzed employing four different algorithms: ΔCt, BestKeeper, NormFinder, and geNorm.

### 2.9 ΔCt method

The ΔCt method compares the relative expression of pairs of genes within each sample to confidently identify suitable control genes (Silver et al., 2006). Briefly, a pair is created with candidate genes A and B. Thereafter, the difference in their Ct values in the same sample is calculated to obtain ΔCt. The standard deviation (SD) of ΔCt for the pair (genes A and B) in all samples is then calculated (A vs. B). This calculation is performed for all pairs containing gene A, such as A vs. C and A vs. D. The arithmetic means of all SD (mean SD) for gene A represent the variation in the expression of gene A with respect to all candidate genes, allowing it to be compared with other genes. Accordingly, the comparisons provide information on pairs showing less variation in expression and hence help in identifying gene(s) with stable expression among the samples evaluated, with the gene with the lowest value being the most stable reference gene. This method does not compromise accuracy and allows non-specialist personnel to perform it by merely repeating a relatively simple calculation (Okamura et al., 2020).

### 2.10 BestKeeper

BestKeeper (Pfaffl et al., 2004) identifies the best housekeeping gene using two evaluation indicators. The first indicator is the SD of the quantification cycle (Cq) value for the expression of each reference gene; the lowest SD value indicates the most stable reference gene. When the SD of a reference gene is large, the variation in the BestKeeper index is also large. In such cases, developers recommend removing candidate genes with an SD > 1. Next, the geometric mean of the reference gene Cq values in each sample is calculated using the BestKeeper index. The Pearson correlation coefficient (r) between the Cq value of each reference gene and the BestKeeper index is used as another index. The highest r-value represents the most stable reference gene. As BestKeeper requires the evaluation of both SD and r, we created a general ranking by considering the geometric mean of the rankings of both indicators.

### 2.11 NormFinder

NormFinder (Andersen et al., 2004) evaluates variations in expression within and between groups. The stability value used in NormFinder reflects within- and between-group variations, and a stable reference gene is evaluated when the value is small. NormFinder analysis requires a large sample, and it is recommended to use at least eight samples per group and 5–10 genes as candidate reference genes to evaluate stability.

### 2.12 geNorm

In the geNorm algorithm (Vandesompele et al., 2002), the average pairwise variation of a particular gene with all other control genes is defined as the internal control gene stability measure, M. The M value indicates the stability of expression, and the lowest value represents the most stable reference gene. When combining two or more reference genes, pairwise variation (Vn/n + 1) is calculated to determine the appropriate number of reference genes to be combined, and Vn/n + 1 < 0.15 is considered an appropriate cutoff value.

### 2.13 Statistical analysis

The expression of each gene is presented as mean ± SD. The Shapiro–Wilk test was used to confirm the normal distribution of the data. When a significant difference was observed in the one-way analysis of variance (ANOVA) with time (in days) as the factor, multiple comparisons were performed using the Bonferroni method. Statistical significance was set at *P* < 0.05. The statistical software IBM SPSS Statistics Version 28.0 for Mac (IBM, Armonk, NY, USA) was used for this analysis.

## 3 Results

**Fig. 1:**
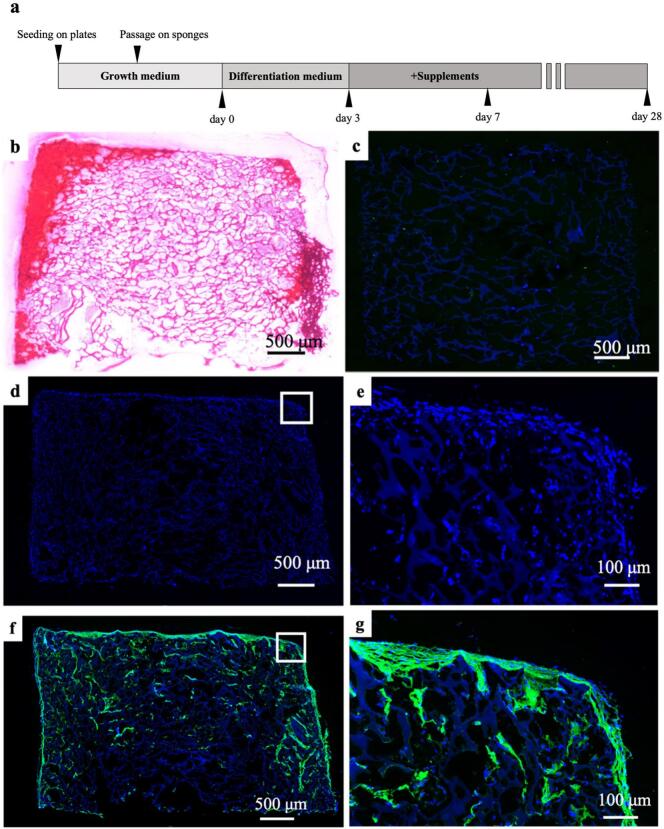
Three-dimensional osteogenic differentiation culture using human induced pluripotent stem cells (hiPSCs). (**a**) Schema showing three-dimensional osteogenic differentiation culture using hiPSCs. (**b**) Sections of the sponge were incubated for 28 days, and then stained with Alizarin Red S. Positive areas can be observed at the edges of the sponge. COL1A1 expression was analyzed using confocal laser microscopy. (**c**) Atelocollagen sponge without seeded hiPSCs. (**d)–(g**) Section of the sponge cultured for 28 days after seeding hiPSCs. Blue fluorescence indicates the nuclei and green fluorescence indicates COL1A1 expression. (**d**), (**e**) Cells stained only with DAPI without staining for COL1A1. (**e**) An enlarged image of a part of (**d**). Both (**d**) and (**e**) show the presence of nuclei at the edges and inside the sponge. (**f**), (**g**) Cells with additional staining for COL1A1. (**g**) An enlarged image of a region from (**f**). COL1A1-positive areas can be observed at the edges and inside the sponge. COL1A1, type I collagen alpha 1; hiPSC, human induced pluripotent stem cell.

hiPSCs were seeded on atelocollagen sponges and cultured for 3D osteogenic differentiation for 28 days (Fig. 1**a**).

### 3.1 Alizarin red S staining

Cross-sections of the atelocollagen sponge collected on day 28 of continued osteogenic differentiation were stained with Alizarin Red S (Fig. 1**b**). Positive Alizarin Red staining was observed at the periphery of the sponge, indicating calcium deposition as a result of osteogenic differentiation. This staining revealed that hiPSCs differentiated into osteoblasts and that mineralization was induced in these cells, as the calcium deposits were stained red (Fig. 1**b**).

### 3.2 Immunostaining

The presence of type I collagen secreted by cells within the atelocollagen sponge was assessed using confocal laser scanning microscopy (Fig. 1**c**, **d**, **e**, **f**, **g**). In comparison with the negative control without cells, the 28-day post-osteogenic induction sections showed areas with positive COL1A1 staining within the periphery and in the interior of the sponge, indicating the presence of type I collagen (Fig. 1**c**, **d**, **e**, **f**, **g**).

### 3.3 SEM

**Fig. 2:**
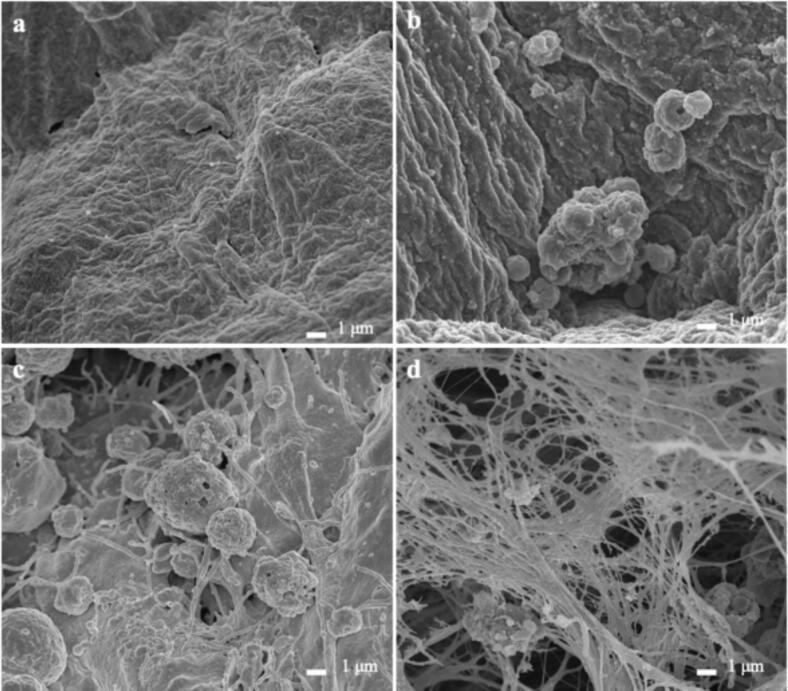
Scanning electron microscopy images of cross-sections of an atelocollagen sponge (magnification: ×5000). (**a**) Cross-section of the sponge without seeded hiPSCs. (**b**) Day 0: hiPSCs adhered to the sponge. (**c**) Day 7: osteoblasts with extending collagen fibers. (**d**) Day 28: collagen fibers filling the voids in the sponge in a three-dimensional manner. hiPSC, human induced pluripotent stem cell.

The cross-section of the atelocollagen sponge was observed at a × 5000 magnification. Fig. 2**a** shows the cross-section of the sponge without seeded cells. After seeding hiPSCs on the sponge and continuing the culture in the maintenance medium, the cells were observed adhering to the cross-section of the sponge on day 0 (Fig. 2**b**). By day 7, the osteoblasts had extended collagen fibers, which are bone matrix proteins (Fig. 2**c**). By day 28, the collagen fibers filled the interior of the sponge in a 3D manner (Fig. 2**d**).

### 3.4 Stability of reference genes for RT-qPCR

**Fig. 3:**
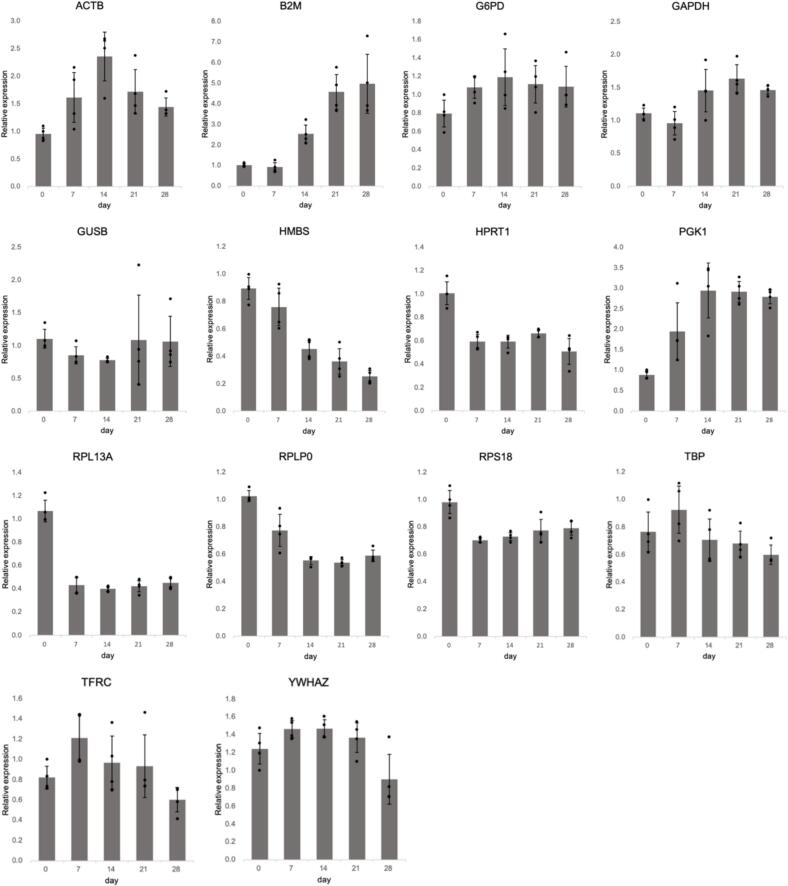
Expression of reference genes during three-dimensional osteogenic differentiation of hiPSCs. Values are presented as mean ± standard deviation from four samples. hiPSC, human induced pluripotent stem cell.

**Fig. 4:**
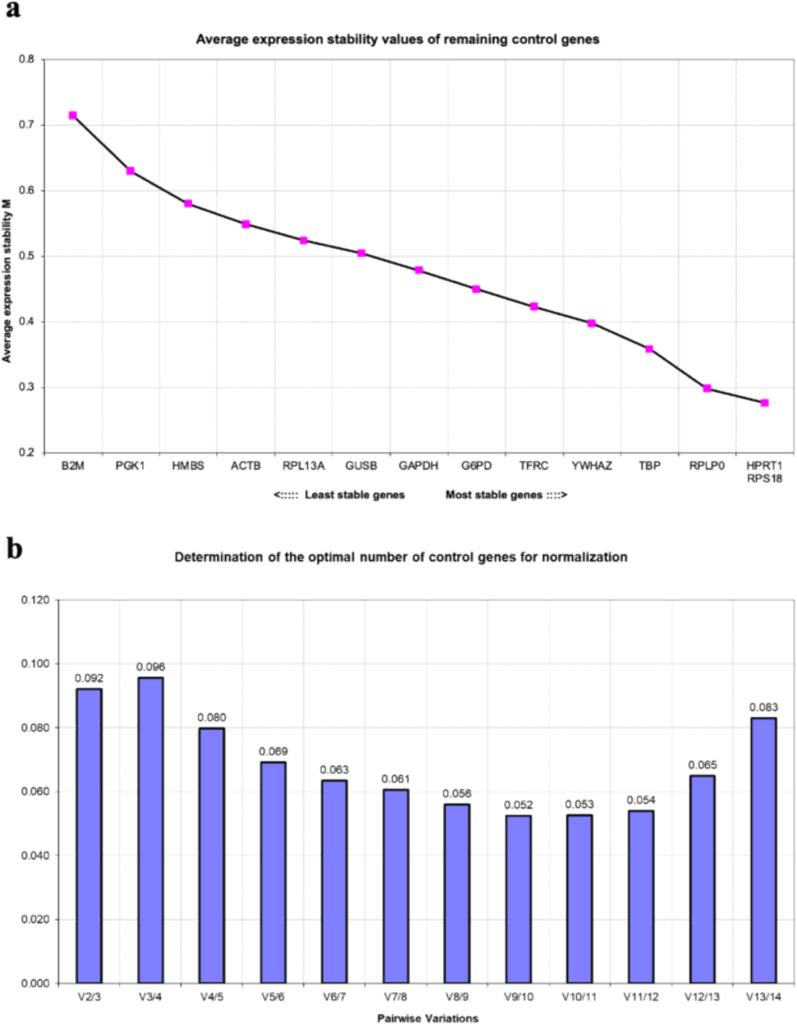
geNorm results. (**a**) Ranking of internal control genes based on mean expression stability (M). (**b**) Optimal number of control genes needed for normalization using pairwise variation (Vn/n + 1). If Vn/n + 1 is <0.15, the number of internal control genes is considered appropriate.

Four samples (technical replicates) were collected on days 0, 7, 14, 21, and 28 to generate a total of 20 samples. RT-qPCR was performed to compare the expression stability of candidate internal control genes (Table 2). Using the threshold cycle (Ct; Fig. 3) and PCR efficiency values from the validation data for each primer, the values presented in Table 3 were calculated. Notably, all Ct values were <35, indicating good RNA quality. In the ΔCT method, the gene with the lowest average standard deviation (SD) was *RPS18*, followed by *TBP*. The highest values were observed for *B2M* and *PGK1*, both of which had an average SD greater than one. In BestKeeper, none of the genes had an average SD greater than one, and hence no gene was excluded. *RPS18* exhibited the lowest average SD, followed by *G6PD*. Conversely, *B2M* had the highest average SD, followed by *PGK1*, which matched the genes with low stability as per the ΔCT method. Additionally, the r-values were limited to 10 genes using the BestKeeper algorithm. Therefore, the top 10 genes based on the average SD were selected, with *TFRC* having the highest value. The top 10 genes were ranked based on a combination of the results from the average SD and r-values, and the geometric means are presented in Table 3. For NormFinder, all hiPSC samples were treated as a single group and induced to undergo osteogenic differentiation. *GAPDH* and *RPS18* showed the lowest stability values, whereas *B2M* had the highest stability value. Fig. 4 shows two graphs created using geNorm. The pair with the lowest M value was *RPS18* and *HPRT1*, with a threshold below 0.5. In contrast, *B2M* exhibited the highest value (Fig. 4**a**). All V2/3-V13/14 values were below 0.15 (Fig. 4**b**). These results indicate that the combination of *RPS18* and *HPRT1* would provide an accurate evaluation of target gene expression in these cells. The general ranking was determined using the geometric mean of the rankings for each of the four methods (Table 3). The most stably expressed genes, from highest to lowest, were *RPS18*, *TBP*, and *HPRT1*. In contrast, *B2M* was the least stable, based on the results of all four methods.

**Table 2:** Primers used for amplifying the target genes in the present study.

Assay	Gene Symbol	Gene Name	Function	Assay ID
PrimePCR SYBR Green Assay (Bio-Rad)	*TBP*	TATA box-binding protein	General RNA polymerase II transcription factor	qHsaCID0007122
	*POU5F1*	POU class 5 homeobox 1	Embryonic stem cell marker	qHsaCED0038334
	*NANOG*	Nanog homeobox	Transcription factor involved in maintaining embryonic stem cell pluripotency	qHsaCED0043394
	*OC*	Osteocalcin	Protein for bone mineralization	qHsaCED0038437
	*SOST*	Sclerostin	Protein that regulates bone formation by osteocytes	qHsaCED0046506
	*PHEX*	Phosphate regulating endopeptidase X-linked	Bone mineralization regulator	qHsaCED0042914
TaqMan Gene Expression Assay (Applied Biosystems)	*RUNX2*	Runt related transcription factor 2	Transcription factor critical for osteoblast differentiation	Hs00231692_m1
	*ALP*	Alkaline phosphatase, liver/bone/ kidney	Enzyme involved in mineralization during bone formation	Hs01029144 m1
	*COL1A1*	Collagen type I alpha 1	Essential for bone structure and strength	Hs00164004_m1

**Table 3:** Expression stability and ranking of candidate reference genes assessed using the ΔCt method, BestKeeper, NormFinder, and geNorm.

Reference gene	ΔCt		BestKeeper					NormFinder		geNorm		Comprehensive ranking	
	Mean SD	Rank	SD	Rank	Coeff. of corr.	Rank	General rank	Stability value	Rank	M value	Rank	Geometric mean of ranks	General rank
*ACTB*	0.71	10	0.415	10	0.399	5	8	0.467	12	0.549	11	10.137	**10**
*B2M*	1.237	14	0.971	14	–	–	14	1.922	14	0.714	14	14.000	**14**
*G6PD*	0.615	4	0.258	2	0.375	6	5	0.216	3	0.450	7	4.527	**5**
*GAPDH*	0.645	7	0.309	6	0.176	9	9	0.135	1	0.478	8	4.738	**7**
*GUSB*	0.707	9	0.341	7	0.364	7	7	0.338	8	0.504	9	8.207	**9**
*HMBS*	0.885	12	0.639	12	–	–	12	0.396	11	0.580	12	11.742	**12**
*HPRT1*	0.609	3	0.267	4	0.512*	4	6	0.245	5	0.276	1	3.080	**3**
*PGK1*	1.002	13	0.742	13	–	–	13	0.872	13	0.630	13	13.000	**13**
*RPL13A*	0.761	11	0.430	11	–	–	11	0.355	10	0.524	10	10.488	**11**
*RPLP0*	0.631	6	0.345	8	0.324	8	10	0.302	6	0.298	3	5.733	**8**
*RPS18*	0.569	1	0.171	1	0.083	10	2	0.143	2	0.276	1	1.414	**1**
*TBP*	0.591	2	0.264	3	0.537*	3	1	0.237	4	0.358	4	2.378	**2**
*TFRC*	0.676	8	0.389	9	0.647*	1	1	0.351	9	0.423	6	4.559	**6**
*YWHAZ*	0.628	5	0.294	5	0.57*	2	2	0.302	6	0.397	5	4.162	**4**

Significance indicated at *P < 0.05.

### 3.5 Expression of each target gene during 3D osteogenic differentiation

**Fig. 5:**
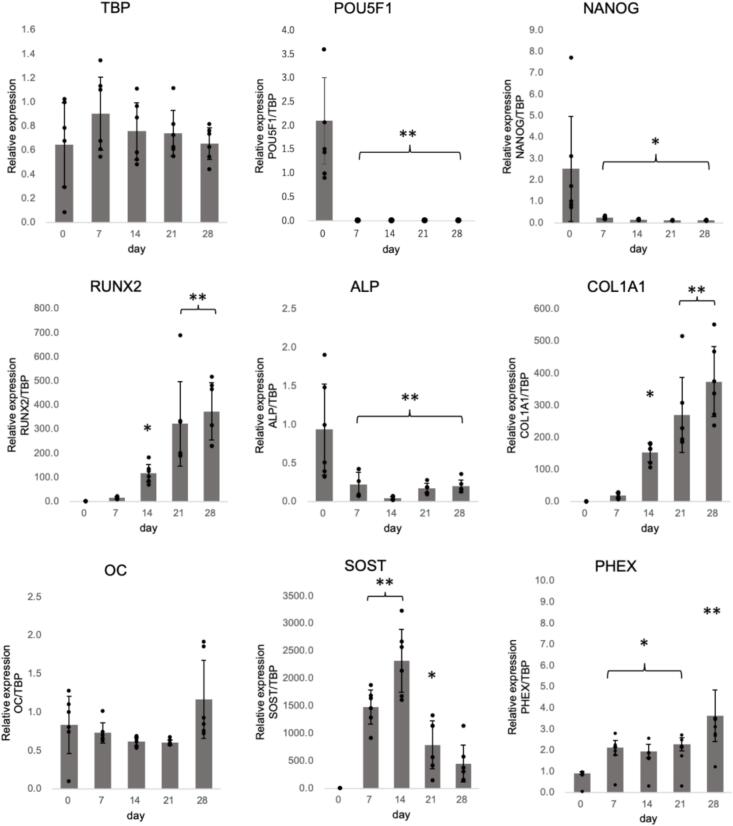
Expression of undifferentiated and osteogenic marker genes during hiPSC osteogenic differentiation (normalized with *TBP* expression). Values are presented as mean ± standard deviation from individual samples; they were analyzed using the one-way analysis of variance (ANOVA) with Bonferroni correction for multiple comparisons; *P < 0.05 and **P < 0.01 indicate statistical significance compared with day 0.

To validate the previous results, RT-qPCR was performed using *TBP* as the reference gene and the relevant primers (Table 2). Similar to the process used for the internal control genes, samples from days 0, 7, 14, 21, and 28 were analyzed. To reduce the variation in differentiation rates between samples, six sponges were sampled each day. The relative expression levels of each gene were calculated using the Ct values obtained from RT-qPCR employing the ΔΔCt method (Fig. 5). All samples had an A260/A280 ratio > 2.0, indicating high RNA purity and none of the samples showed a Ct value >35. Fig. 5 shows the changes in the relative expression levels of the reference gene *TBP* and markers for undifferentiated, osteoblast, and osteocyte differentiation states up to day 28 of bone differentiation. The expression did not change significantly throughout the study period. The expression of *POU5F1* and *NANOG*, which are markers of the undifferentiated state, was significantly downregulated from day 0 to 7. The expression of the osteoblast differentiation markers *RUNX2* and *COL1A1* gradually increased from day 0 and showed a significant increase on day 21. The expression of *OC* also showed a similar increasing trend on day 28. *ALP*, which is a marker of osteoblast differentiation and is also expressed in osteoprogenitor cells, was initially highly expressed on day 0, indicating the presence of undifferentiated cells. However, its expression decreased with time and showed an increasing trend again on day 28, owing to osteoblast differentiation. SOST is a protein expressed in mature bone cells; the expression of the gene encoding it was significantly increased on day 7. The expression of *PHEX*, which is also expressed in bone cells, showed a significant increase on day 7 compared with that on day 0 and was markedly increased on day 28. These results indicate that the hiPSCs cultured in this study successfully differentiated into osteoblasts and osteocytes in a 3D environment. Through normalization with the expression of *TBP*, we could assess the loss of the undifferentiated state in these cells. Furthermore, the assessment of the expression of endogenous control genes, which was the primary objective of this study, was conducted during the induction of osteogenic differentiation in hiPSCs.

## 4 Discussion

The MIQE guidelines emphasize the need to standardize the expression of target genes using stably expressed reference genes to correct for variability between samples in terms of RNA extraction yield, reverse transcription efficiency, and PCR efficiency. This highlights the importance of selecting appropriate reference genes for each tissue or cell type and for individual experiments. This also ensures the reliability and accuracy of the gene expression data obtained through RT-qPCR experiments, as it corrects for variations introduced during different steps of the experimental process, from sample preparation to data analysis. The choice of suitable reference genes is a critical aspect of experimental design in qPCR (Bustin et al., 2009). Unfortunately, most studies related to osteogenic differentiation rely on commonly used reference genes, such as *GAPDH* (Go et al., 2017; Drynda et al., 2018; Chen et al., 2019) and *18S* rRNA (Czekanska, et al., 2014), without proper validation of their suitability for standardization. As a result, research attempting to identify optimal reference genes is limited, and to the best of our knowledge, no such genes have been identified in the context of 3D osteogenic differentiation culture experiments using hiPSCs.

In this study, to obtain reliable results, we employed four algorithms, namely, the ΔCt method, BestKeeper, NormFinder, and geNorm, to identify reliable reference genes for hiPSCs. Reference gene validation based on these four algorithms has been reported by several studies on various cell types and experimental conditions (Gao et al., 2019; Bednarz-Misa et al., 2020; Bantulà et al., 2022; Mu et al., 2023). Jacob et al. (Jacob et al., 2013) recommended the use of reference genes selected using three or more stability algorithms. RefFinder (http://www.heartcure.com.au/reffinder/ or https://blooge.cn/RefFinder/) is a web-based tool that, upon input of Ct values, automatically runs the algorithms of BestKeeper, NormFinder, geNorm, and the comparative ΔCt method. It calculates stability rankings and an overall ranking based on each algorithm (Xie et al., 2023). RefFinder's results have been widely used in many studies (Taïhi et al., 2016; De Spiegelaere et al., 2015). In this study, RefFinder identified RPS18, TBP, and HPRT1 as the most stable reference genes, consistent with our findings. Therefore, RefFinder may simplify the complex task of identifying optimal reference genes by allowing easy execution of each algorithm.

We identified the most stable reference genes among the 14 endogenous control genes in the context of 3D osteogenic differentiation culture experiments using hiPSCs. Overall, we confirmed that *RPS18*, *TBP*, and *HPRT1* were the most stable reference genes. However, we did not use *RPS18* as an internal reference gene. This is because according to previous literature, the expression of *RPS18* is exceptionally high and considerably deviates from the Ct values of most target genes. Additionally, there is an imperfect correlation between its rRNA and mRNA expression levels because rRNA transcription is carried out by different RNA polymerases (Quiroz et al., 2010; Kozera and Rapacz, 2013). Therefore, we believe that *RPS18* should not be considered a suitable reference gene for future studies. Our results are consistent with those of Hasler et al. (2020), who reported that despite showing stable expression in 3D culture, *RPS18* may not be a suitable reference gene. We ranked the most stable reference genes in the following order: *TBP* > *HPRT1* > *YWHAZ*. Rauh et al. (Rauh et al., 2015) conducted a 3D osteogenic differentiation culture with BM-MSCs and validated 31 endogenous control genes using geNorm and NormFinder. They found that *TBP*, *TFRC*, and *HPRT1* were the most stably expressed reference genes. Our results are consistent with their findings; we found that *TBP* and *HPRT1* showed stable expression. Hasler et al. (2020) verified the optimal reference genes from eight endogenous control genes in 3D osteogenic differentiation using BM-MSCs with geNorm, NormFinder, and ΔCt method. They reported that *PPIA*, *OAZ1*, and *GUSB* were the most suitable reference genes for normalization, with *TBP* ranking fifth. From these findings, it is apparent that even in reference gene validation experiments in the context of 3D osteogenic differentiation, the choice of reference genes should be considered carefully each time because the results can vary from one experiment to another.

In a previous study, we found that *TBP*, *TFRC*, and *RPLP0* were the most stable reference genes in a 28-day 2D osteogenic differentiation culture experiment (Okamura et al., 2020). This finding suggests that *TBP* is a reliable and stably expressed reference gene in two as well as 3D osteogenic differentiation of hiPSCs. Brinkhof et al. (Brinkhof et al., 2018) used the same four algorithms that we used to investigate reference genes in human MSCs in both 2D and 3D cultures. Combining the results from the 2D and 3D cultures, they concluded that *TBP*, *PPIA*, and *TFRC* were the most stable reference genes, which is consistent with our findings. Hasler et al. (2020) conducted 3D osteogenic differentiation experiments using BM-MSCs; they identified *PPIA*, *OAZ1*, and *GUSB* as the top three stably expressed reference genes in 3D culture and *RPLP0*, *GAPDH*, and *PPIA* as exhibiting stable expression in 2D culture. This finding indicates that even when using the same cell type, careful consideration is needed for selecting reference genes, especially when culture dimensions differ (2D vs. 3D).

Currently, 3D cultures are gaining attention, and it is important to consider that the choice of the scaffold material may affect the selection of reference genes. Hasler et al. (2020) conducted 3D osteogenic differentiation experiments using human BM-MSCs with a methacrylated hyaluronic acid hydrogel as the scaffold. Rauh et al. (2015) used peracetic acid-treated human cancellous bone cubes as the scaffold and obtained entirely different results for suitable reference genes. Additionally, Chooi et al. (2013) investigated the stability of reference genes in rabbit chondrocytes cultured in alginate and agarose matrices. They reported *TBP*, *RPL5*, and *RPL18* as the most stable reference genes in the alginate scaffold and *TBP* and *HPRT1* as the most stable in the 3D agarose matrix. These findings underscore the need for careful consideration when selecting reference genes, as the choice of scaffold material can influence the decision.

In the present study, *B2M* was identified as the least stable gene, which is consistent with the results of previous studies (Okamura et al., 2020). Additionally, commonly used reference genes, such as *ACTB* and *GAPDH*, ranked 10th and 7th, respectively, showing intermediate to low stability, making them inappropriate for use as reference genes. De Jonge et al. (2007) conducted a meta-analysis of 13,629 human gene arrays and found that commonly used reference genes (*B2M*, *ACTB*, and *GAPDH*) were not among the top 50 most stable reference genes. Brinkhof et al. (2018) concluded that *B2M* was the most unstable gene in both 2D and 3D cultures of MSCs. Panina et al. (2018) suggested that *ACTB* should not be considered a stable reference gene when performing experiments related to cell stemness because its expression is influenced by the experimental conditions. Furthermore, Stürzenbaum and Kille (2001) recommended the use of *ACTB* when tissues undergo extensive morphological changes. Li et al. (2015) reported *GAPDH* to be the least stable reference gene among the candidate reference genes, and its sensitivity has been shown to be affected by the 3D environment (Rauh et al., 2015). Therefore, it is advisable to avoid the conventional use of *B2M*, *ACTB*, and *GAPDH* as reference genes.

Based on changes in the relative expression levels of markers for the undifferentiated, osteoblast, and osteocyte stages up to day 28, we noted that hiPSCs underwent osteogenic differentiation on an atelocollagen sponge scaffold into osteoblasts and osteocytes. Furthermore, Alizarin Red S staining, fluorescent immunostaining, and SEM revealed that osteoblasts and osteocytes derived from hiPSCs synthesized collagen fibers and calcified matrices, inducing 3D bone formation. From a biomaterial perspective, this finding suggests that atelocollagen sponges provide a conducive environment that supports bone formation from hiPSC-derived osteoblasts and osteocytes. These findings provide new insights into bone tissue regeneration.

Despite presenting some convincing findings, this study has some limitations. First, during the culture period of 28 days, fluctuations were noted in the expression of *SOST*, an osteocyte differentiation marker, indicating that the differentiation stages of hiPSCs were not clearly defined during this period. Additionally, the specific signaling pathways involved in this process remain unclear. Nevertheless, it should be noted that the primary objective of this study was to identify optimal reference genes in 3D osteogenic differentiation cultures, which was achieved. Detailed investigation of these aspects should be undertaken in the future using RNA sequencing. Second, we only examined a limited set of 14 candidate reference genes. It is possible that other unexplored reference gene candidates exhibit even more stable expression. This opens up possibilities for obtaining more precise and accurate RT-qPCR results. Third, we did not examine different hiPSC lines. Therefore, it is unclear whether TBP is the best reference gene for different hiPSC lines. However, we believe that it is crucial to evaluate the reference gene for each case, as the optimal reference gene may differ between experiments, even when using the same cell line. Therefore, we consider that there is no universally applicable reference gene that can be used across different hiPSC lines. Fourth, although we directly obtained osteogenic progenitor cells, it may be ideal to induce a gradual differentiation process from mesodermal or ectodermal cells to mesenchymal cells and ultimately to osteoblasts.

## 5 Conclusions

In the emerging field of regenerative medicine, identifying the most suitable source of stem cells and cell lines is of utmost importance for optimizing patient treatment. The initial stages of evaluating stem cell properties are critical, and any inaccuracies at this stage can potentially affect patient treatment prospects. There is no universally suitable gene for normalization, and therefore, an internal reference gene must be optimized for the standardization of gene expression under different experimental conditions. Based on the results of the present study, we propose considering *TBP* as a potential reference gene candidate in hiPSC osteogenic differentiation studies. It is also advisable to confirm and use the most appropriate reference genes for experiments under different conditions.

## CRediT authorship contribution statement

**Masakazu Okamoto:** Data curation, Formal analysis, Investigation, Methodology, Project administration, Writing – original draft. **Yusuke Inagaki:** Funding acquisition, Project administration, Supervision, Writing – review & editing. **Kensuke Okamura:** Conceptualization, Formal analysis, Methodology, Validation, Visualization, Writing – review & editing. **Yoshinobu Uchihara:** Writing – review & editing. **Kenichiro Saito:** Writing – review & editing. **Akihito Kawai:** Data curation, Writing – review & editing. **Munehiro Ogawa:** Writing – review & editing. **Akira Kido:** Supervision. **Eiichiro Mori:** Conceptualization, Funding acquisition, Writing – review & editing. **Yasuhito Tanaka:** Supervision, Writing – review & editing.

## Funding

This work was supported by JSPS KAKENHI [grant numbers JP18K1663 and 20 K09508 to Y.I.]; 10.13039/100009619AMED [grant number JP23wm0425004 to E.M.]; Japan Sports Medicine Foundation, 2020 [to K.O.]; and The Japanese Orthopaedic Society of Knee, Arthroscopy and Sports Medicine, 2020 [to K.O.].

## Declaration of competing interest

E.M. is the founder–CEO of molmir, Inc. The other authors have nothing to declare.

## Data Availability

The datasets generated and analyzed during the current study are available from the corresponding author on reasonable request.
